# Hepatic sclerosed hemangioma with special attention to diffusion-weighted magnetic resonance imaging

**DOI:** 10.1186/s40792-017-0414-z

**Published:** 2018-01-03

**Authors:** Tatsunori Miyata, Toru Beppu, Kunitaka Kuramoto, Shigeki Nakagawa, Katsunori Imai, Daisuke Hashimoto, Tomohiro Namimoto, Yo-ichi Yamashita, Akira Chikamoto, Yasuyuki Yamashita, Hideo Baba

**Affiliations:** 1 Department of Gastroenterological Surgery, Graduate School of Life Sciences, Kumamoto University, 1-1-1, Honjo, Chuo-ku, Kumamoto, 860–0811 Japan; 2 Department of Surgery, Yamaga City Medical Center, 511, Yamaga, Kumamoto, 861–0593 Japan; 3 Department of Diagnostic Radiology, Faculty of Life Sciences, Kumamoto University, 1-1-1, Honjo, Chuo-ku, Kumamoto, 860–0811 Japan

**Keywords:** Sclerosed hemangioma, Diffusion-weighted sequences of magnetic resonance imaging, Apparent diffusion coefficient

## Abstract

**Background:**

A hepatic sclerosed hemangioma (HSH) is a very rare benign liver tumor. The correct preoperative diagnosis of HSH is very difficult because its features of imaging are similar to those of intrahepatic cholangiocarcinoma or colorectal liver metastasis.

**Case presentation:**

We experienced five patients who were diagnosed histologically with HSH. The preoperative diagnoses were HSH in two patients, cavernous hemangioma in one, intrahepatic cholangiocarcinoma in one, and colorectal liver metastasis in one. All patients were treated with hepatectomy (one laparoscopic and four laparotomies), and the diagnosis was completed by histological investigation of the resected specimen. In particular, we investigated the apparent diffusion coefficient (ADC) mean value using diffusion-weighted sequences of magnetic resonance imaging (DW-MRI). The average of the ADC mean (ADC_mean_) value of HSH was 1.94 × 10^−3^ mm^2^/s (range 1.73–2.10 × 10^−3^ mm^2^/s), which was higher than the value of common malignant liver tumors. Interestingly, the ADC_mean_ values were almost the same between the degenerate (1.90 ± 0.17 × 10^−3^ mm^2^/s) and the non-degenerate areas (1.95 ± 0.26 × 10^−3^ mm^2^/s) in HSH.

**Conclusions:**

The ADC_mean_ value seemed to be quite useful to preoperatively distinguish HSH from other malignant liver tumors.

**Electronic supplementary material:**

The online version of this article (10.1186/s40792-017-0414-z) contains supplementary material, which is available to authorized users.

## Background

A hepatic sclerosed hemangioma (HSH) is a very rare benign subtype of hepatic hemangioma and is detected in only 0.2% of cases in a study of 1000 consecutive necropsies [1]. Since its image features resemble those of hepatic malignancies such as intrahepatic cholangiocarcinoma (ICC) [2] or colorectal liver metastasis (CRLM) [3], HSH is frequently suspected to be hepatic malignancies that need to be resected. Overall, HSH is often diagnosed correctly after resection. To avoid an unnecessary operation, accurate preoperative diagnosis of HSH is required.

One of the most effective methods for the differential diagnosis of liver tumors is magnetic resonance imaging (MRI). Conventional and diffusion-weighted MRI (DW-MRI) are effective techniques for the characterization of focal solid hepatic lesions [4]. In addition, the apparent diffusion coefficient (ADC) value in DW-MRI has been useful for distinguishing malignant from benign liver tumors [4–6]. To the best of our knowledge, this is the first study about the utility of the ADC mean (ADC_mean_) value in multiple patients with HSH. We herein would like to demonstrate detailed imaging findings of five cases along with literature reviews.

## Cases presentation

### Patients and imaging methods

From July 2009 to November 2016, five patients in our institutions were histologically diagnosed using a resected specimen with HSH, which were confirmed by pathologists. The patients underwent imaging examinations, including ultrasonography (US), contrast-enhanced computed tomography (CT), gadolinium-ethoxybenzyl-diethylenetriamine pentaacetic acid (Gd-EOB-DTPA)-enhanced MRI, and ^18^F-fluorodeoxyglucose positron-emission tomography (FDG-PET). We considered the part with the contrast effect in CT as non-degenerative site, and the other part as degenerative site. To obtain an accurate preoperative diagnosis, we additionally performed DW-MRI with respiratory triggering using *b* values of 0 and 800 s/mm^2^, and ADC maps were generated using *b* values of 0 and 800 s/mm^2^ for calculation of the ADC value. Of the five patients, four were evaluated using a 3.0-Tesla whole-body MRI scanner and one patient was evaluated using a 1.5-Tesla whole-body MRI scanner; studies have demonstrated similar ADC values between the 1.5- and 3.0-Tesla scanners [7, 8]. The ADC_mean_ value was calculated by taking the average of the six areas of ADC values, which were randomly selected at the degenerative (three areas) and non-degenerative areas (three areas) of each HSH. Similarly, we separately evaluated the ADC_mean_ values in the degenerative and non-degenerative areas. For the comparative analysis, we also evaluated the ADC_mean_ values in the degenerative and non-degenerative areas of the adenocarcinoma with necrosis in the liver. We randomly selected six ICC patients and four CRLM patients from our database of patients with liver tumors. They were also diagnosed histologically using the resected specimen. The radiological data was independently assessed by two radiologists. We also retrospectively investigated the preoperative blood test data, imaging data, and pathological findings.

### Statistical analysis

Comparisons between the ADC_mean_ values of the degenerative and non-degenerative areas were examined using Student’s *t* test. The results with two-tailed values of *P* < 0.05 were considered to be statistically significant. All statistical analyses were performed using JMP software (Version 12; SAS Institute, Cary, NC, USA).

### Patient characteristics

The clinical characteristics of the patients with HSH were summarized in Table 1. Four male and one female were included, and their mean age was 60 years (range 34–79 years). Hepatitis B virus surface antigen (HBs-Ag) was positive in two patients, and hepatitis C virus antibody (HCV-Ab) was negative in all patients. All patients’ carcinoembryonic antigen and carbohydrate antigen 19–9 were within their normal ranges. The most predicted preoperative diagnoses were HSH in two patients, cavernous hemangioma in one, ICC in one, and CRLM in one.

**Table 1:** Characteristics of five patients with hepatic sclerosed hemangioma

Case	Age	Gender	Etiology	CA19-9 (U/ml)	CEA (ng/ml)	Comorbidity	Location	Size (mm)	Preoperative diagnosis	Operation
Case 1	79	M	None	8.5	1.0	None	S5/6	43	ICC	Anatomical resection
Case 2	34	F	None	16	1.1	None	Whole right lobe	148	Hemangioma	Extended hemi-right hepatectomy
Case 3	70	M	HBV	6.4	2.5	Colon cancer	S6	15	CRLM	Laparoscopic partial hepatectomy
Case 4	63	M	HBV	12.7	1.7	HCC (S4)	S7	6	Sclerosed hemangioma	Partial hepatectomy
Case 5	54	M	None	9.1	1.4	None	S8	27	Sclerosed hemangioma	Partial hepatectomy

*HBV* hepatic B virus, *CA19-9* carbohydrate antigen 19-9, *CEA* carcinoembryonic antigen, *ICC* intrahepatic cholangiocarcinoma, *CRLM* colorectal liver metastasis

### US, CT, and PET imaging features

**Fig. 1:**
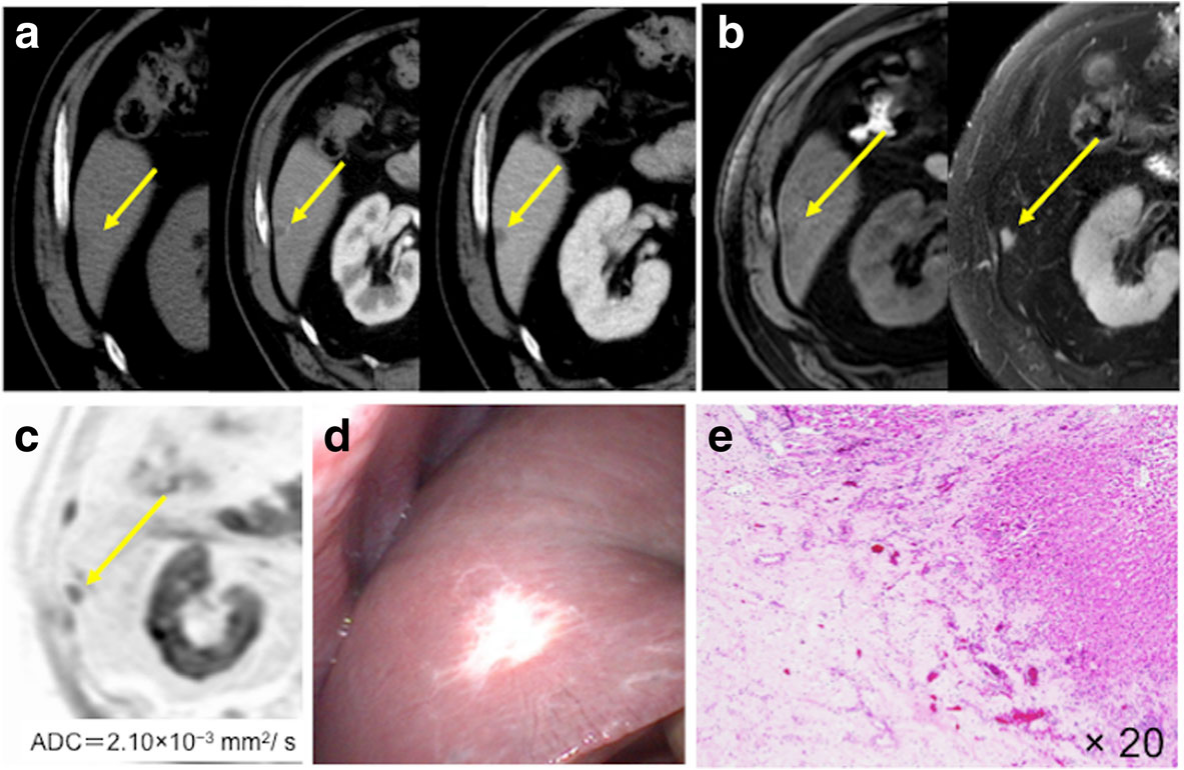
The tumor showed ring enhancement in the arterial phase and low-density mass in the portal phase (**a**) in a CT image. MRI showed that HSH is a hypointense mass on a fat-suppressed T1-weighted image and a hyperintense mass on a T2-weighted MRI scan (**b**). The ADC_mean_ value of the mass was 2.10 × 10^−3^ mm^2^/s on DW-MRI (**c**). A whitish tumor was shown on the liver surface and capsular retraction was identified (**d**). Histologically, there were many small vessels with fibrous replacement and hyalinization (**e**)

**Fig. 2:**
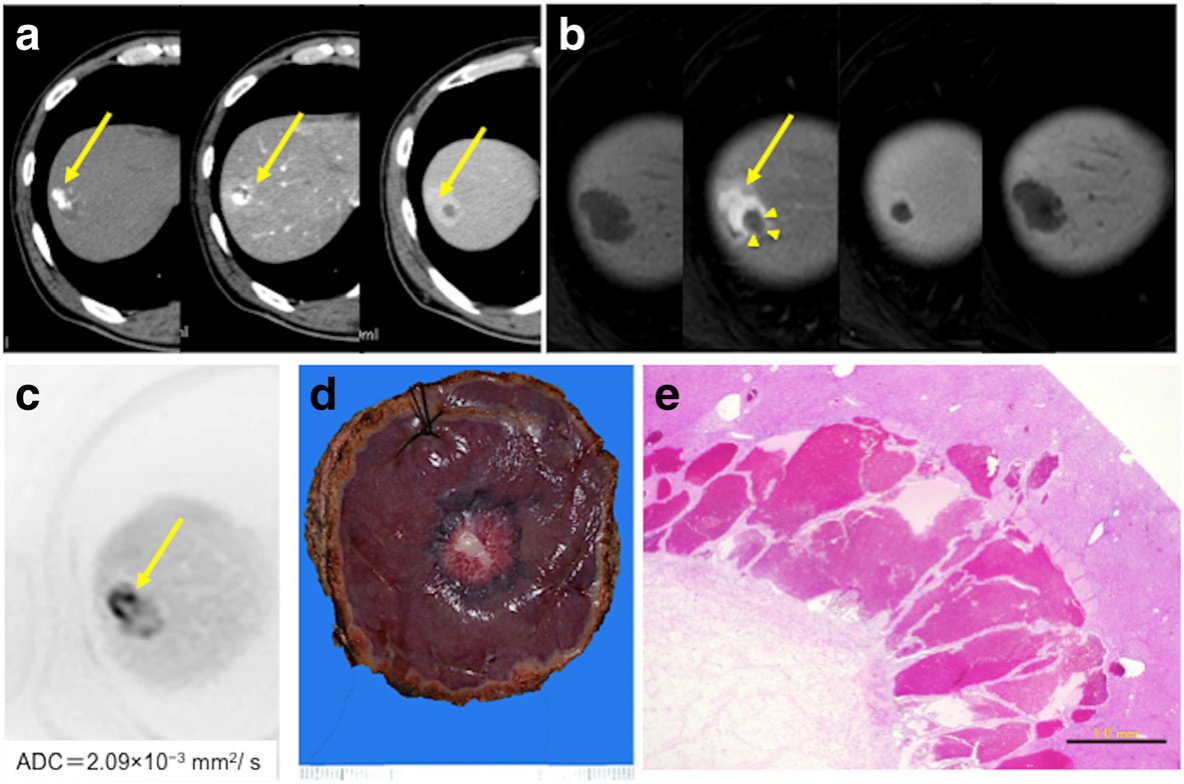
The tumor was seen as a mass with peripheral nodular enhancement in the arterial phase and progressive centripetal fill-in in the portal phase of CT imaging (**a**). MRI showed that HSH was a hypointense mass on a fat-suppressed T1-weighted image and a mass with peripheral nodular enhancement in the early phase. In the late phase, the tumor was an iso-hypointense mass and filling defect in the hepatocyte phase of dynamic MRI. We considered the part with the contrast effect in CT or MRI as non-degenerative site (arrow) and the other part as degenerative site (arrow head). (**b**). The ADC_mean_ value of the mass was 2.09 × 10^−3^ mm^2^/s on DW-MRI (**c**). The whitish part was shown within the hemangioma-like tumor on the liver surface and capsular retraction was identified (**d**). Histologically, there were many small vessels with fibrous replacement and hyalinization. The scale bar shows 1.0 mm (**e**)

HSH presented with different echogenicity based on the degree of regenerative tissue in the ultrasound images. The features of HSH on CT were summarized in Table 2. All tumors were solitary with lobulated shapes, and the tumor sizes varied from 6 to 148 mm. Three patients had simultaneous hemangiomas, and three patients showed contractive changes on the tumor surface. All tumors had low densities on plain CT images. Two patients had ring enhancements, and two patients had peripheral nodular enhancements in the arterial phase. Two tumors had a low-density mass, and three had a progressive centripetal fill-in pattern from the portal to venous phase in the CT images (as seen in cases 3 and 5 shown in Figs. 1a and 2a). Three patients underwent FDG-PET; however, no FDG accumulation was observed in the HSH.

**Table 2:** Computed tomography (CT) features of five patients with hepatic sclerosed hemangioma

					Dynamic CT		
Case	Simultaneous hemangioma	Capsular retraction	Arterioportal shunt	Plain CT	Arterial phase	Portal phase	Venous phase
Case 1	+	−	+	Low	Ring enhancement	Progressive centripetal fill-in	Progressive centripetal fill-in
Case 2	−	−	−	Low	Peripheral nodular enhancement	Progressive centripetal fill-in	Progressive centripetal fill-in
Case 3	+	+	−	Low	Ring enhancement	Low	Low
Case 4	−	+	+	Low	Low	Low	Low
Case 5	+	+	+	Low	Peripheral nodular enhancement	Progressive centripetal fill-in	Low

### MRI features

**Fig. 3:**
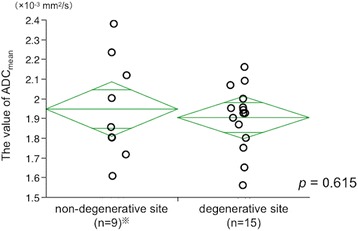
The average values of ADC_mean_ were almost the same between the degenerate (1.90 ± 0.17 × 10^−3^ mm^2^/s) and the non-degenerate areas (1.95 ± 0.26 × 10^−3^ mm^2^/s) (*t* test, *p* = 0.615). ^※^Two cases were very small tumors; therefore, we could not determine the ADC_mean_ value in the non-degenerative area

**Fig. 4:**
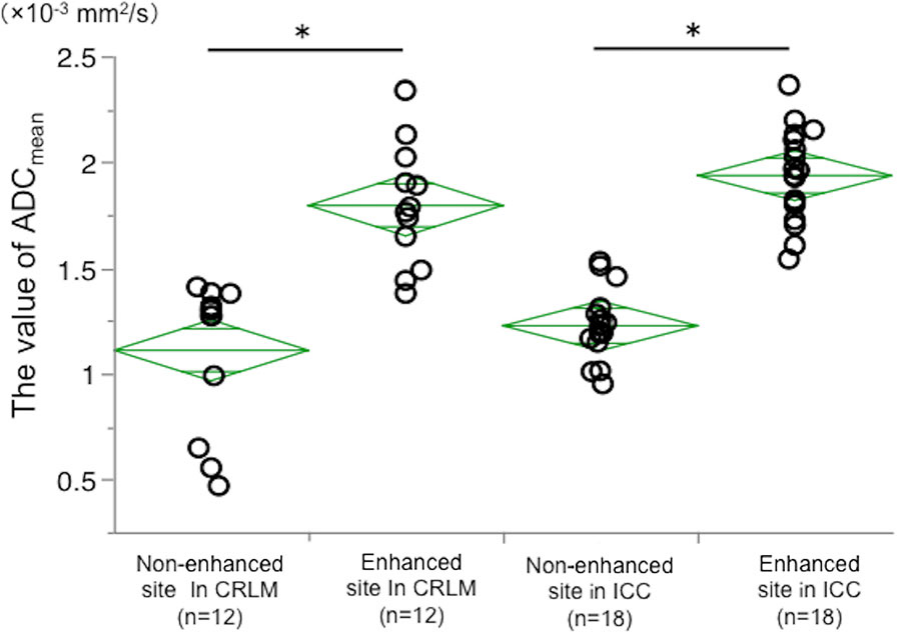
The average values of ADC_mean_ in six ICCs (18 area) and four CRLMs (12 area) were significantly different between the degenerate and the non-degenerate areas (*t* test, *p* < 0.0001). Asterisk denotes *p* < 0.0001

The features of HSH on MRI were summarized in Table 3. All tumors had no fatty tissue and were hypointense on T1-weighted images and hyperintense on T2-weighted images, as seen in case 3 (Fig. 1b). Three tumors showed ring enhancements, and two tumors showed peripheral nodular enhancements in the arterial phase. Three tumors showed progressive centripetal fill-in patterns during the late phase. All tumors were described as defects in the hepatocyte phase on MRI, as seen in case 5 (Fig. 2b). The average ADC_mean_ value for the five cases was 1.94 × 10^−3^ mm^2^/s (range 1.72–2.09 × 10^−3^ mm^2^/s). The average (± standard deviation) values of the ADC_mean_ were approximately the same between the degenerate (1.90 ± 0.17 × 10^−3^ mm^2^/s) and non-degenerate areas (1.95 ± 0.26 × 10^−3^ mm^2^/s; *p* = 0.615) (Fig. 3). However, there were significant differences in the ADC_mean_ values between the enhanced (1.90 ± 0.25 × 10^−3^ mm^2^/s) and non-enhanced areas (1.18 ± 0.25 × 10^−3^ mm^2^/s) of ICC and CRLM in the early phase of MRI (*p* < 0.0001) (Fig. 4). Their background factors were listed in Additional file 1: Table S1.

**Table 3:** Magnetic resonance imaging (MRI) features of five patients with hepatic sclerosed hemangioma

Case	Border	Fatty tissue	T1-weighted T2-weighted	Dynamic MRI findings				ADC mean (×10^–3^ mm^2^/s)	
				Fat-suppressed T1-weighted	Early phase	Late phase	Hepatocyte phase	Degenerative area	Non-degenerative area
Case 1	Lobulated shape	–	Hypo Hyper	Hypo	Ring enhancement	Progressive centripetal fill-in	Defect	1.65	1.80
								1.56	1.61
								1.75	2.00
Case 2	Lobulated shape	–	Hypo Hyper	Hypo	Peripheral nodular enhancement	Progressive centripetal fill-in	Defect	1.87	1.80
								1.80	1.86
								1.93	1.72
Case 3	Lobulated shape	–	Hypo Hyper	Hypo	Ring enhancement	Progressive centripetal fill-in	Defect	2.07	–
								2.16	
								2.09	
Case 4	Lobulated shape	–	Hypo Hyper	Hypo	Ring enhancement	Hypo	Defect	2.00	–
								1.93	
								1.96	
Case 5*	Lobulated shape	–	Hypo Hyper	Hypo	Peripheral nodular enhancement	Iso-hypo	Defect	1.94	2.23
								1.95	2.12
								1.90	2.38

*ADC* apparent diffusion coefficient, *Hypo* hypo intensity, *Hyper* hyper intensity

*The results were obtained using 1.5-tesla MRI

### Operation and pathological examination

Although three patients were diagnosed with suspicious benign tumors, we could not deny their possibility of malignancy. Furthermore, patients desired to receive hepatectomy to obtain pathological diagnosis. We underwent laparoscopic hepatectomy for one (case 3) and hepatectomy with laparotomy for the others (Table 1). Histologically, there were many small vessels with fibrous replacement and hyalinization in all cases. All patients could discharge without any complications.

## Discussion

Herein, we described the clinical, pathological, and imaging features of five patients with HSH. Interestingly, the ADC_mean_ values had a different pattern between HSH and malignant liver metastases; however, the ADC_mean_ values were similar in the degenerate and non-degenerate areas of HSH. To the best of our knowledge, the current study is the first report to mention the differences of ADC_mean_ values between the degenerate and non-degenerate areas in HSH.

Cavernous hemangioma is the most common hepatic vascular tumor in adults [9], while HSH is an extremely rare liver tumor [1]. HSH has various degenerative changes such as extensive fibrosis with subsequent hyalinization, marked narrowing or obliteration of the vascular spaces, and hemorrhage or sclerosis [10]. Makhlouf and Ishak compared the findings of HSH and cavernous hemangioma in terms of pathology. Compared with cavernous hemangioma, HSH contained abundance of collagenous tissue and elastic fibers around and between small sclerotic vessels. In addition, vascular endothelial markers (i.e., CD31, CD34, FVIII-R Ag) were weaker, suggesting there was senescence in blood vessels [11]. However, the mechanism for degenerative changes in HSH and the origin of HSH remain unclear at present. HSH itself is benign tumor; therefore, it is considered good to follow up without resection as long as there are no symptoms.

Cavernous hemangioma is usually found as a single mass tumor of five cm or less [12]. It is contrasted from the periphery in dynamic CT, the contrast effect gradually spreads to the center (progressive centripetal fill-in), and furthermore, the contrast effect prolonging from the equilibrium phase to the delay phase (prolonged enhancement) is recognized [13]. On the other hand, HSH has several different imaging features. Doyle et al. reported that the findings of geographic pattern, capsular retraction, decreased size over time, and loss of previously seen regions of enhancement suggested HSH. Moreover, they also showed a difference in the transient hepatic attenuation, rim enhancement, and nodular regions of intense enhancement, as seen in typical hemangiomas [14]. In our cases, all tumors showed ring enhancement or peripheral nodular enhancement in the arterial phase and two cases were described as having progressive centripetal fill-in from the portal to the venous phase in CT images. Therefore, all cases had previous features of HSH or cavernous hemangioma; however, we could not deny the possibility of a malignancy because ICC and CRLM have several similar features on imaging [2, 3]. In particular, in case 3, the patient was simultaneously diagnosed with colon cancer, which contributed to a misdiagnosis of the liver tumor as CRLM. Sakamoto et al. reported that liver hemangioma of 5 cm or less was frequently diagnosed as malignant tumor [15], and even in our cases, four cases were HSH of 5 cm or less. Thus, it is quite difficult to perform accurate diagnosis only with dynamic CT or MRI.

The ADC_mean_ value on DW-MRI may be useful for distinguishing HSH from other malignant liver tumors. In malignant tumors, we often observed tissue invasion and a cancerous environment with increased cellularity and enlarged cells, which contributed to a reduction in the extracellular space. In addition, cancer cells harbored more hyperchromatin and exhibited a high nucleus-to-cytoplasm ratio, limiting the diffusion of water molecules in intracellular spaces [4, 5]. These histopathological characteristics result in a decrease in the ADC_mean_ value. Bruegel et al. showed that the ADC_mean_ value was 1.22 × 10^−3^ mm^2^/s for metastatic liver tumors and 1.92 × 10^−3^ mm^2^/s for hemangiomas [16]. Namimoto et al. also showed that the ADC_mean_ values of the hepatocellular carcinoma, metastases, ICC, and hemangiomas were 1.15 ± 0.21 × 10^−3^, 1.23 ± 0.32 × 10^−3^, 1.52 ± 0.26 × 10^−3^, and 2.09 ± 0.43 × 10^−3^ mm^2^/s, respectively [5]. In addition, Hida et al. showed that the ADC_mean_ value of HSH was 2.01 × 10^−3^ mm^2^/s [17]. Therefore, these authors suggested that the ADC_mean_ values of HSH and cavernous hemangioma (approximately 2.00 × 10^−3^ mm^2^/s) tended to be higher than those of malignant liver tumors. In our five cases, the average ADC_mean_ value of the five HSHs was 1.94 × 10 × 10^−3^ mm^2^/s. This result support previous reports.

Interestingly, our results also showed the ADC_mean_ values were almost of the same degree and higher than malignant tumors between the degenerate and the non-degenerate areas, suggesting that the non-degenerate areas had a similar cellular density compared to the degenerate areas in HSH. In fact, in the pathological findings, although the fibrous tissue of the degenerate area was abundant, the cell density in the degenerate area was not very high. In addition, it is recognized that the size of HSH decreases; it is rare to limit the extracellular space. On the other hand, in the case of a malignant tumor, tissue invasion and cancer nests exhibiting increased cellularity and enlarged cells. This reduced the size of the extracellular space, and the cancer cells harbored more organelles, enlarged nuclei, and hyperchromatism and exhibited a high nuclear-to-cytoplasmic ratio, limiting the diffusion of water molecules in intracellular spaces [5]. Therefore, there is a difference in the ADC value between the contrast part and the non-contrast part in malignant tumor. Thus, our data suggested that it was possible to distinguish between HSH and malignant liver tumors by calculating the ADC_mean_ values in tumor and by comparing both the degenerate and non-degenerate areas in HSH.

## Conclusions

Using the ADC_mean_ value on DW-MRI may be an effective method for distinguishing HSHs from other malignant liver tumors, especially in liver adenocarcinomas with degenerative areas. Further investigation of a large number of HSH patients will be needed.

## Additional file

Additional file 1: Table S1. Characteristics of patients with ICC or CRLM. (DOCX 142 kb)

## Abbreviations

ADC: Apparent diffusion coefficient
CRLM: Colorectal liver metastasis
CT: Computed tomography
FDG-PET: Fluorodeoxyglucose positron-emission tomography
Gd-EOB-DTPA: Gadolinium-ethoxybenzyl-diethylenetriamine pentaacetic acid
HSH: Hepatic sclerosed hemangioma
ICC: Intrahepatic cholangiocarcinoma
MRI: Magnetic resonance imaging
US: Ultrasonography
