# Supplementary material for: Hepatic sclerosed hemangioma with special attention to diffusion-weighted magnetic resonance imaging
Source: Surg Case Rep. 2018 Jan 3;4:3. doi: 10.1186/s40792-017-0414-z (PMC5752647; doi:10.1186/s40792-017-0414-z)
Supplement: Supplementary file 1 [file 40792_2017_414_MOESM1_ESM.docx]

Supplementary Table 1 Characteristics of patients with ICC or CRLM

Case	Age	Gender	Etiology	CA19-9 (U/ml)	CEA (ng/ml)	Size (mm)	Differentiation	ADC mean ($\times 10^{-3}$ mm^2/s)	
								Enhanced area	Non-enhanced area
ICC 1	79	M	None	0.1	1.8	82	moderate	1.54<br>1.61<br>1.82	1.17<br>1.31<br>1.20
ICC 2	58	F	None	231.3	6.8	75	moderate	2.06<br>2.20<br>2.37	1.51<br>1.53<br>1.46
ICC 3	66	F	None	44.5	4.1	80	moderate	1.70<br>1.82<br>1.73	1.19<br>1.24<br>1.19
ICC 4	63	F	None	21.4	2.1	85	moderate	1.80<br>1.94<br>1.97	1.01<br>0.95<br>1.01
ICC 5	73	F	None	53373	2102	72	moderate	1.93<br>2.02<br>1.96	1.23<br>1.26<br>1.15
ICC 6	74	M	None	61.6	2.2	90	moderate	2.11<br>2.13<br>2.15	1.19<br>1.20<br>1.28
CRLM 1	74	M	None	32.7	18.5	47	moderate	1.65<br>1.74<br>1.76	0.56<br>0.65<br>0.47
CRLM 2	64	F	None	6.1	15.5	85	well	1.89<br>1.79<br>1.90	1.32<br>1.32<br>0.99
CRLM 3	57	F	HCV	39.8	7.8	53	moderate	1.38<br>1.44<br>1.49	1.38<br>1.41<br>1.27
CRLM 4	65	M	None	55.8	148.7	58	moderate	2.34<br>2.13<br>2.02	1.28<br>1.30<br>1.38

Abbreviation: ICC, intrahepatic cholangiocarcinoma; CRLM, colorectal liver metastasis; HCV, hepatic C virus; CA19-9, carbohydrate antigen 19-9; CEA, carcinoembryonic antigen
